# Rare Occurrence of Herpes Zoster of Trigeminal Nerve following Extraction of Tooth

**DOI:** 10.1155/2015/891618

**Published:** 2015-12-27

**Authors:** A. Winnifred Christy, T. Jones Raja Deva Thanmbi, J. Leelavathy, Antoinette Rhema Louis

**Affiliations:** ^1^Department of Oral Medicine and Radiology, C.S.I College of Dental Sciences and Research, Madurai 625001, India; ^2^C.S.I College of Dental Sciences and Research, Madurai 625001, India

## Abstract

Herpes Zoster also known as Shingles is an acute viral infection which is an extremely painful and incapacitating ailment. It results from the reactivation of the varicella zoster virus. The triggering factors for the onset of an attack of Herpes Zoster include some form of immunosuppression. The diagnosis of Herpes Zoster can be made on proper medical history and a thorough clinical examination. Here is the report of a male patient affected by Herpes Zoster infection which followed after extraction of a lower first molar.

## 1. Introduction

Herpes Zoster is a painful acute infectious viral disease caused due to the inflammation of dorsal root ganglia or extramedullary cranial nerve ganglia, leading to vesicular eruptions of the skin or mucous membrane in an area supplied by the affected nerve. The most commonly affected dermatomes are the thoracic (45%), cervical (23%), and trigeminal (15%) [1, 2].

The primary infection of varicella zoster virus (VZV) is the chicken pox and, due to the characteristic of latency of the herpes group of viruses, VZV gets reactivated to cause Herpes Zoster infection in later age.

The incidence of latent Herpes Zoster increases 5–10-fold after the age of 80 years [3].

## 2. Case Report

A male patient of 49 years reported to the Department of Oral Medicine and Radiology, C.S.I. College of Dental Sciences and Research, with the chief complaint of pain and ulceration on left side of face and mouth for four days. Patient gave the history of extraction of the tooth 5 days ago after which multiple vesicles formed on the left side of the face and inside the mouth which made him uncomfortable while ingesting food. He had visited a dentist who diagnosed the condition as angioedema and prescribed antihistamines for three days but they did not alleviate any of the symptoms and the pain and ulcerations worsened. He did not have any relevant medical history. He is not a smoker but occasionally consumes alcohol.

**Figure 1:**
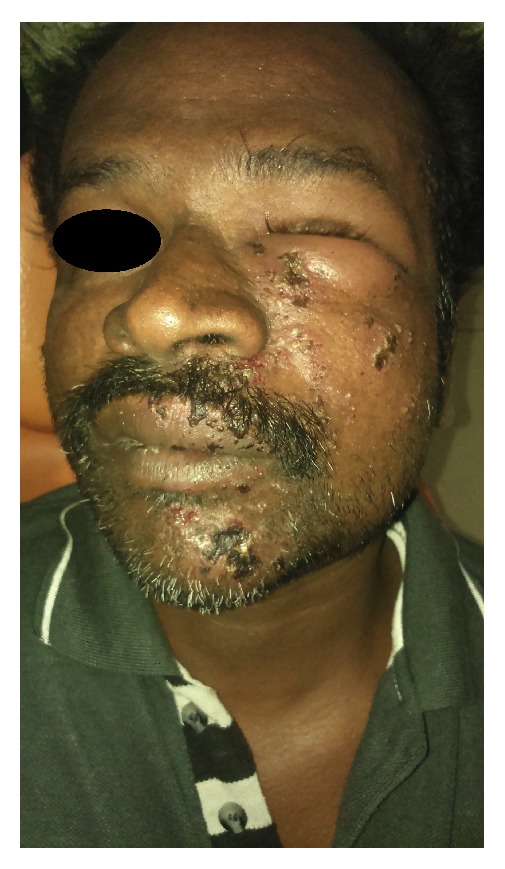
Vesicles showing unilateral distribution extraorally.

**Figure 2:**
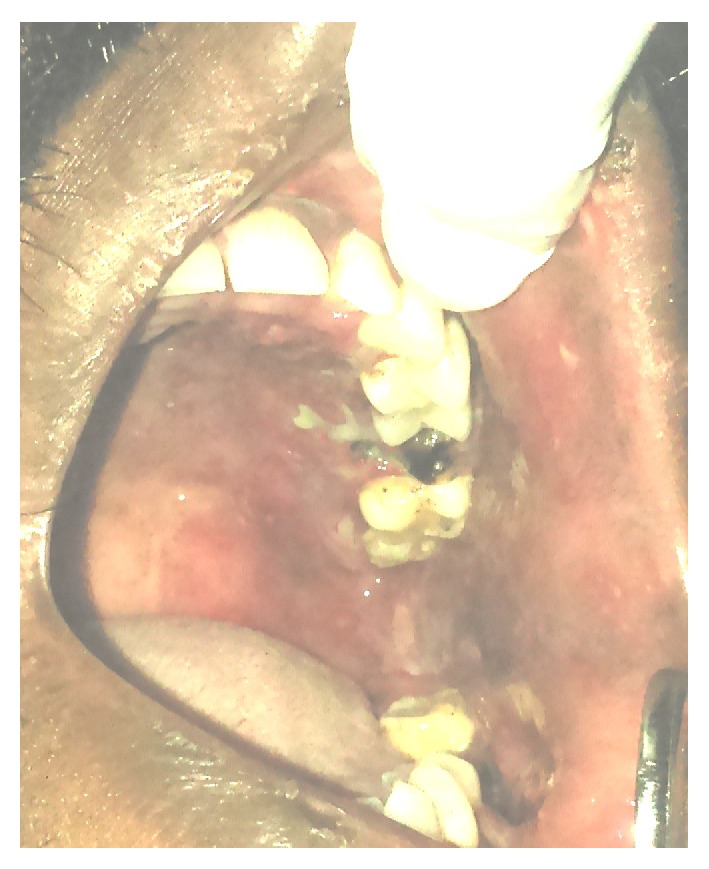
Erosions on palate with necrosis and sloughing in 26 region.

On examination, unilateral multiple vesicles with few of them ulcerated were found on the left side of the face and facial asymmetry due to diffuse swelling which extended superiorly to the upper eyelid was evident (Figure 1). On intraoral examination, unilateral multiple ulcerations were evident on the left side of hard and soft palate which did not cross the mid line (Figure 2).

**Figure 3:**
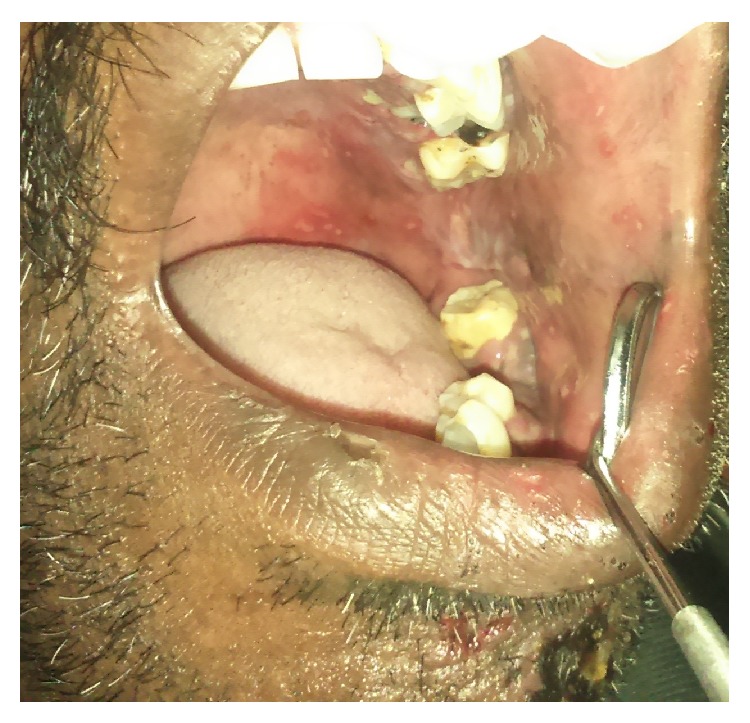
Vesicles and erosions seen in the buccal mucosa, mandibular alveolar mucosa, and palate.

Ulcerations were also evident on left buccal mucosa and alveolar mucosa in relation to 36 (Figure 3). The ulcers were covered with slough and bleeding on slightest provocation was evident. The ulcers were extremely tender on palpation.

Patient was subjected to few investigations to rule out any immunocompromised status. The hemogram and serum glucose levels were within normal limits. ELISA for HIV was negative. Patient was referred to a general physician for hydration and ophthalmic evaluation. A dose of antivirals and steroids which included Valacyclovir 1000 mg 3 times a day and Prednisolone 20 mg 3 times a day and topical acyclovir for 1 week was prescribed.

**Figure 4:**
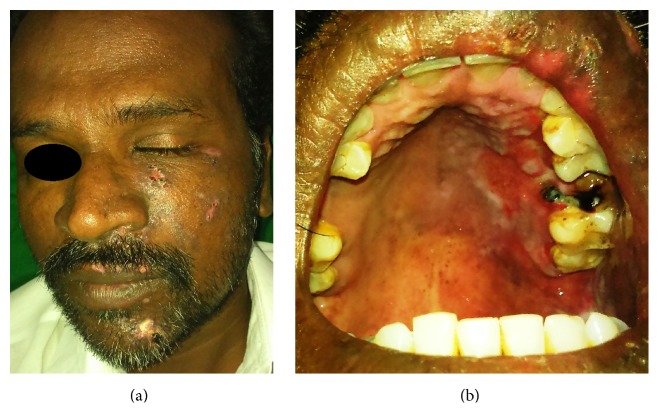
After one week of treatment with antivirals and steroids.

The patient responded to antivirals and steroids well within a week and showed considerable healing. Patient was evaluated every week and steroids were tapered over four weeks and stopped. Gradual healing was observed in phases (Figures 4(a) and 4(b)).

**Figure 5:**
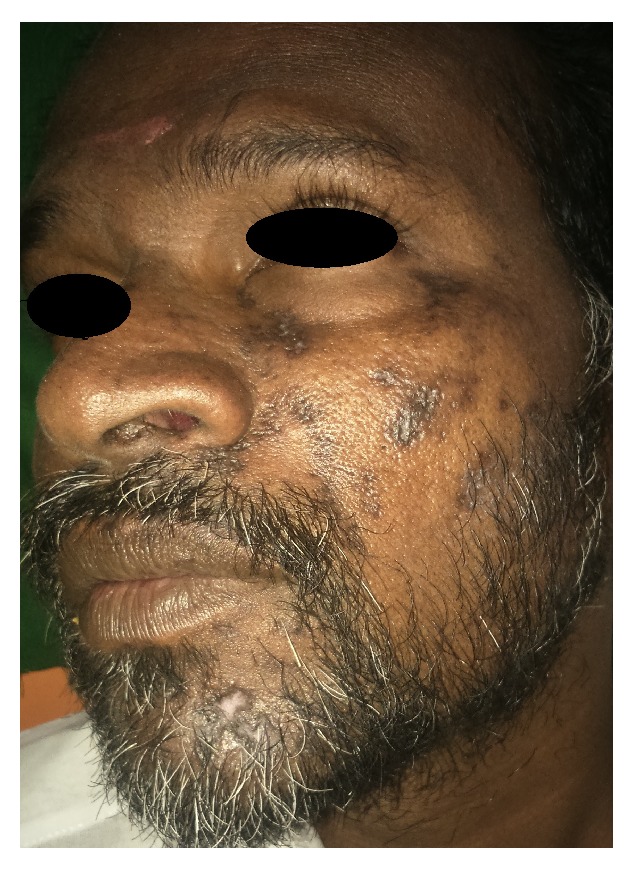
Healed ulcers extraorally after a month.

**Figure 6:**
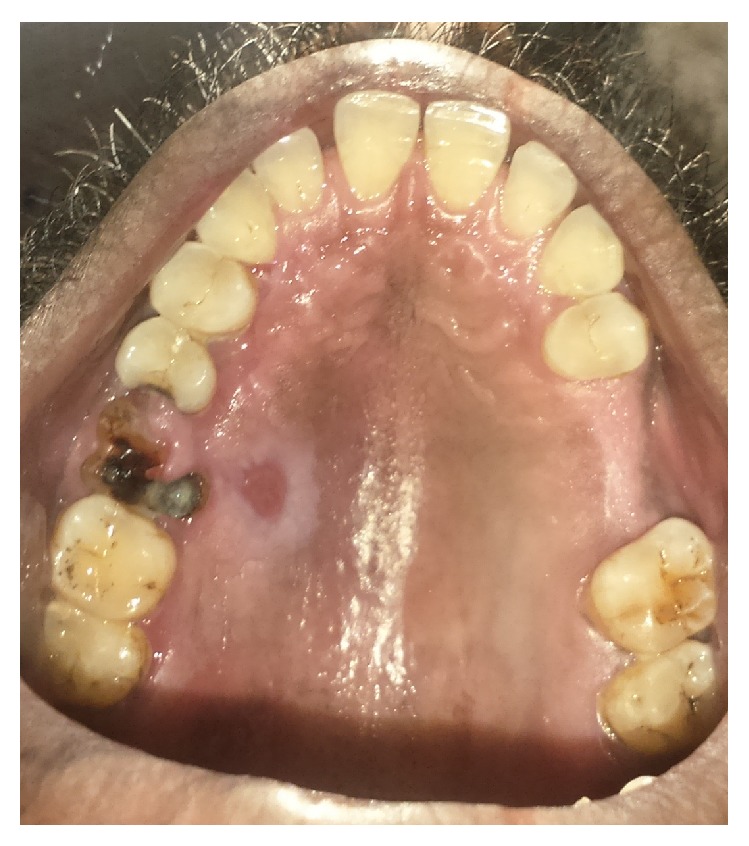
Intraoral healed ulcers after a month.

By fourth week the patient was seen only with scars on the left side of face (Figures 5 and 6).

Systemic steroids could have prevented complications as it has been 3 months and the patient is apparently healthy at present.

## 3. Discussion

Herpes Zoster has an estimated life time incidence of 10–20% and gets reactivated with some form of immunosuppression. Herpes Zoster infection is common in elder persons, HIV-positive individuals, and patients affected by malignant blood dyscrasias or malignant tumours or undergoing immune suppressive therapy and radiotherapy [4].

The most noted point of our case report was that there was no previous history of any herpetic simplex infection in childhood or recurrent herpes labialis in later stage. It is believed that the patient could have contracted chicken pox early in his life as the incidence of chicken pox in a tropical country like India is very high. Also the unilateral distribution of the erosions and the lesions pertaining only to the oral and maxillofacial region with involvement of ophthalmic, maxillary, and mandibular division of trigeminal nerve is suggestive of Herpes Zoster rather than a herpes simplex infection. The infection was triggered by a traumatic extraction of left mandibular lower first molar. El Hayderi et al. postulated that HSV reactivation occurs during surgical procedures involving trigeminal nerve in 50% of patients and an anaesthetic block may irritate the nerve leading to reactivation and recrudescence of herpes lesion [5]. Two cases of Herpes Oticus and a similar case of Herpes Zoster after extraction of tooth have been reported [6, 7]. This case was reported to the department with a delay as he had previously consulted a local dentist, who was unable to diagnose the condition, and the patient was treated with antihistamines.

Involvement of the second and third branches of the trigeminal nerve results in vesicular lesions in oral cavity. The vesicular lesions develop 2–4 days after prodromal period of fever, weakness, fatigue, and stiffness of the neck [8]. Our patient did not have any typical prodromal symptoms. Characteristic signs of oral HZ are the presence of unilateral vesicles that break rapidly, leaving small ulcers. On skin and lips, vesicle ruptures can result in erosions covered by pseudomembranes and haemorrhagic crusts which were also seen in our patient. By the end of second or third week the crusts and pseudomembranes disappear with eventual healing of the vesicular lesions [3, 9]. A frequent complication of HZ infection is development of postherpetic neuralgia (PHN) within one to three months of healing of VZ lesions and is characterised by pain, paresthesia, hypoesthesia, or allodynia and can persist for months and years. This patient was followed up for up to six months and did not develop postherpetic neuralgia.

The duration of healing of Herpes Zoster lesions and the severity of pain associated with the disease have been shown to be considerably less with prompt administration of antiviral agents. However these benefits have been found in patients who received antiviral agents within 72 hours after the onset of the rash [4]. But our patient reported to us only after 5 days of having eruptions due to misdiagnosis. Even after prescription of antivirals and steroids, a delayed healing was noted.

## 4. Conclusion

The early diagnosis of Herpes Zoster and prompt treatment can avoid further complications. Herpes infection following extractions has been reported very rarely. Herpes Zoster infection must also be considered as one of the complications after extraction. The patients must be asked to report to the dentist if there is any symptoms of the disease after extraction. The patient should be under medication and periodically reviewed. Misdiagnosis should be avoided as far as possible unlike this case where herpes infection was misdiagnosed as allergy.
